# A Framework of Joint Energy Provisioning and Manufacturing Scheduling in Smart Industrial Wireless Rechargeable Sensor Networks

**DOI:** 10.3390/s18082591

**Published:** 2018-08-07

**Authors:** Yixiong Feng, Yong Wang, Hao Zheng, Shanghua Mi, Jianrong Tan

**Affiliations:** State Key Laboratory of Fluid Power and Mechatronic Systems, Zhejiang University, Hangzhou 310027, China; fyxtv@zju.edu.cn (Y.F.); wangyong_phd@zju.edu.cn (Y.W.); 11725079@zju.edu.cn (S.M.); egi@zju.edu.cn (J.T.)

**Keywords:** SIWRSNs, energy provisioning, manufacturing scheduling, smart factory

## Abstract

Energy provisioning is always a crucial problem restricting the further development and application of smart industrial wireless sensor networks in smart factories. In this paper, we present that it is necessary to develop smart industrial wireless rechargeable sensor networks (SIWRSNs) in a smart factory environment. Based on the complexity and time-effectiveness of factory operations, we establish a joint optimization framework named J-EPMS to effectively coordinate the charging strategies of wireless sensors and the scheduling plans of machines running. Then, we propose a novel double chains quantum genetic algorithm with Taboo search (DCQGA-TS) for J-EPMS to obtain a suboptimal solution. The simulation results demonstrate that the DCQGA-TS algorithm can maximally ensure the continuous manufacturing and markedly shorten the total completion time of all production tasks.

## 1. Introduction

With the rise of Industry 4.0 and smart manufacturing, smart industrial wireless sensor networks (SIWSNs) have become a vitally important part of smart factories. Manufacturing under smart factories gives access to actively acquiring production information, real-time analyzing the production status, and intelligently executing production decisions [1]. In order to accurately obtain process information in a timely manner, a large number of industrial sensors, such as acceleration sensors, vibration sensors, and displacement sensors, need to be deployed in smart factories and form industrial sensor networks. For a long time, the industrial sensor networks mainly adopted the wired field bus technology, involving wired nodes and Industrial Ethernet [2]. With the huge improvement in the level of information and automation of manufacturing plants, the wired industrial sensor networks have given rise to many intricate problems, including difficult regional wiring, limited network expansion, inconvenient nodes migration, and not applying for rotating parts [3]. At the same time, due to the fact that the wired sensors are hard to be mounted on the moving workpieces, all machines and workpieces are unable to form a self-organized system in smart factories. For these reasons, owing to the rapid development of wireless communication technology, wireless sensors have begun to partially take the place of wired sensors mounted on the mechanical devices or moving workpieces. After sensing the manufacturing process information, these wireless sensors will timely transmit data to external servers relying on the local wireless networks. In fact, hybrid industrial sensor networks, including both wireless and wired, have become the key component of information architecture in smart factories [4,5].

Industrial practices have proved that the large-scale applications of industrial wireless sensors not only enhance the data collection capabilities of industrial sensor networks, but also accelerate the smart transformation processes of manufacturing plants [6]. Especially for the digital and intelligent upgrading of some old machines, the use of wireless sensors can avoid the trouble of secondary wiring. Meanwhile, wireless sensors can be mounted on moving workpieces to realize a portable dynamic configuration, which greatly enlarges the range of sensor applications and reduces the difficulty and cost of smart transformations. However, as far as the current application scenarios are concerned, energy provisioning is still a major bottleneck restricting the further development and application of industrial wireless sensor networks (IWSNs) [7]. Most of the traditional industrial wireless sensors are powered by non-rechargeable batteries. The battery capacity and work intensity determine the service life of the sensor. Once the battery is completely consumed, the sensor will permanently fail unless a new battery is replaced. In response to this problem, researchers in related fields have studied how to reduce energy consumption by optimizing the node layout, information fusion, and routing strategies of wireless sensor networks to maximize the lifetime of entire sensor networks [8]. In essence, these methods are mainly to reduce the quantity of sensors, data collection frequency, computational complexity, and network traffic as much as possible. Different from some common scenarios, in order to accurately monitor the production process in real-time, the industrial sensors must keep up high-intensity work for a long time and continuously feed manufacturing data back to the controllers or actuators. For example, while a machine tool is working, some associated displacement sensors must monitor the position of cutters in real-time and send out these data in a timely manner. Due to the machining continuity and processing variability of industrial monitoring targets, sensors associated with the machine often need to keep up a long high-load working time, and their continuous working current may reach more than 20 mA [9]. As a result, industrial wireless sensors often need to be equipped with large capacity batteries, which in turn will lead to a sharp increase in the overall weight of the sensor, and bring some trouble in the layout and selection of wireless sensors, especially in some high-precision manufacturing processes [10]. Beyond that, for the purpose of improving the reliability of data collection, many industrial sensors are highly integrated with the machine, or the deployment location is relatively narrow, and replacing batteries is often difficult to accomplish. In addition, with the aim of reducing the computing load of the central server and enhancing the real-time handling capability of production process data, edge computing technology will be massively adopted by smart factories in the near future [11,12]. Sensor nodes will undertake more single-point computing tasks in a smart factory, which obviously increase the unit energy consumption of sensors. Therefore, by reason of the imperfection of energy provisioning, the limited battery capacity means it is difficult to meet the requirement of continuous processing for a long time, and smart factories are restricted in the lifetime of IWSNs.

To implement energy replenishment in IWSNs, many researchers have made use of wireless charging technology to develop a new kind of sensor network called a Wireless Rechargeable Sensor Network (WRSN), which are considered to ultimately solve the energy provisioning problem of wireless sensor networks [13]. In recent years, wireless charging technology has made a great advance, and some industrial applications have been realized, such as charging for electric toothbrushes, Bluetooth headsets, mobile phones, etc. Existing wireless charging technology solutions mainly include inductive coupling, electromagnetic radiation, and magnetic resonant coupling [14]. Correspondingly, the energy replenishment technologies for wireless rechargeable sensor networks can be divided into two major aspects. One is to study how to use energy capture technology to actively harvest various energy sources from nature to charge the battery, such as solar energy. This method has extremely limited energy provisioning per unit time and is greatly restricted by the spatial and temporal distribution of energy in nature, and is not applicable to a smart factory environment. The other research being done is to monitor the battery status of sensor nodes and to make use of mobile chargers (MCs) to replenish energy of low-energy sensor nodes in a timely manner. This type of energy replenishment is less affected by external environmental factors and is suitable for smart factories. As a result, in order to realize the large-scale applications of industrial wireless sensors, smart factories should establish a stable and reliable wireless rechargeable sensor network, and make use of mobile chargers to supplement energy for low-power sensors in time.

As far as we know, there is little research focusing on how to effectively schedule mobile chargers to replenish energy for smart industrial wireless rechargeable sensor networks (SIWRSNs) in a smart factory environment. In fact, this problem urgently needs to be solved to reliably operate a smart factory. Some scholars have highlighted the importance of the energy replenishment for industrial wireless sensor networks, but they did not extensively study the feasible charging strategies with consideration of the complex factory operations [8]. Compared to wireless sensor networks in other fields, i.e., forest monitoring [15], industrial wireless sensor networks must meet the requirements of continuous sensing, complex computing, and real-time communication for a long time in a smart factory. Once the machine begins to work, the measurement frequency of sensor nodes is usually at the millisecond level, the energy consumption per unit time is relatively high, and unpredictable node failures are absolutely not allowed. In this case, supposing that the battery capacity is fixed, the wireless charging strategies are closely correlated with the actual machining time of the equipment. Furthermore, according to Reference [16], the efficiency of wireless charging can be immensely influenced by the distance and angle between mobile chargers and sensor nodes. At the same time, the charging time of a single sensor node varies greatly with the battery capacity and remaining energy, and the frequency band of wireless charging may interfere with the accuracy of data acquisition and wireless communication of sensor nodes [17]. Hence, with the aim of not affecting the normal operation of factory equipment, smart factory operators should keep the charging process of wireless sensors and the associated machining process in a separate state.

In addition, minimizing the completion time of entire production tasks is a very important goal for factory operations in a smart factory [18]. Correspondingly, in order to ensure controllable manufacturing activities, a reliable production plan must be pre-arranged and rigorously executed, and unpredictable events should be prevented, such as unexpected interruptions caused by sensor failures [3]. So, for the purpose of effectively completing production tasks in a smart factory, it is necessary for factory operators to collaboratively consider the charging strategies and manufacturing scheduling for making an optimal production plan. Therefore, this paper is intended to build a framework of Joint Energy Provisioning and Manufacturing Scheduling (J-EPMS) in SIWRSNs to obtain the optimal production plan. In view of the computation complexity of the problem, we design a corresponding approximate algorithm, and verify the feasibility of the algorithm by numerical simulations. The simulation results show that with the goal of minimizing the total completion time of the entire production tasks, the sensors charging and associated machines operating can be reasonably planned and separately executed.

The remainder of this paper is organized as follows. Section 2 introduces previous work related to the aforementioned WRSNs. Section 3 thoroughly discusses the problem studied in this paper and provides some model assumptions. Section 4 develops the framework of Joint Energy Provisioning and Manufacturing Scheduling (J-EPMS) in smart industrial wireless rechargeable sensor networks and proposes a novel algorithm named DCQGA-TS for J-EPMS. Section 5 shows some simulation results based on DCQGA-TS. Finally, the discussion and conclusions are presented in Section 6.

## 2. Related Work

The rapid development of wireless charging technology provides an effective solution for the energy provisioning problem of wireless sensor networks. In order to collect the object information for a long time, wireless rechargeable sensor networks are being extensively studied in some fields, such as forest monitoring [15], coal mines monitoring [19], civil infrastructure health monitoring [20], and smart grid monitoring [21]. The industrial wireless sensor networks are also receiving more and more attention [3]. Because smart factories are still a rising research field, smart wireless sensors are becoming an indispensable part of modern manufacturing plants and how to charge WRSNs in a smart factory environment remains to be further studied. Learning from these current research results, we can better carry out our research work. In recent years, some researchers have carried out a series of studies on wireless rechargeable sensor networks. By extensively reading these studies, four main aspects can be summarized: the scheduling policies of mobile chargers, the layout design of sensor nodes, network architecture in special scenarios, and the joint optimization in complex scenarios.

First of all, quantity of chargers, scheduling algorithms, and movement paths are focused on in the scheduling policies of mobile chargers. Xu et al. proposed using a single charging vehicle with separable charger array to charge a WRSN [22]. Dai et al. investigated the minimum mobile chargers problem (MinMCP) in a large-scale WRSN [23]. Porta et al. studied an energy harvesting-aware sensor-mission assignment distributed algorithm [24]. Chen and Feng et al. proposed a hop-based mobile charging policy to minimize the number of mobile chargers in a large-scale WRSN [25,26]. Because these mobile chargers are often deployed on the automated guided vehicles (AGVs), researchers paid attention to reducing the number of mobile chargers in the case of ensuring the coverage. In a smart factory, in view of mobile chargers being handheld and low-cost, optimizing the number of mobile chargers is not very important. For scheduling algorithms, Ye et al. studied a kind of replenishing energy algorithm that maximized the charging utility of sensors [27]. Gao et al. studied the optimal scheduling problem with maximal optimal quality of monitoring (QoM) [28]. Lin et al. developed a hybrid clustering charging algorithm in a large-scale WRSN [29]. Zhong et al. proposed a real-time on-demand charging scheduling scheme with limited mobile charger capacity [30]. Shih et al. studied a coverage-aware recharging scheme for WRSNs [31]. Shu et al. put forward a joint energy provisioning and operation scheduling policy to maximize the lifetime of WRSNs [32]. Chang et al. developed a multi-module charging strategy to maximize the lifetime of WRSNs [33]. Because of the high randomness of the energy consumption of sensor nodes, scheduling algorithms are very complex. Different from these, the energy consumption of sensors can be approximately estimated in the case of a given production plan. The complexity of charging for wireless sensors in a smart factory environment is to avoid the simultaneous occurrence of the sensors charging and associated machines operating. Furthermore, Wang et al. developed a novel mobile data gathering framework with vehicle movement costs and capacity constraints [34]. Arivudainambi et al. developed the Daubechies wavelet algorithm to find the optimal position of mobile chargers [35]. Li et al. studied the cooperative deployments of chargers and sink stations [36]. Shu et al. studied how to control the moving velocity of mobile chargers for maximizing the charged energy of nodes [37]. Yang et al. presented an improved grid-based joint charging and routing method to achieve path planning for a mobile charger [38]. Fu et al. presented a novel energy-synchronized mobile charging protocol [39]. Due to the wireless sensors being mounted on different kinds of machines in a workshop, optimizing the movement paths of charging for wireless sensors is not very necessary in a smart factory environment.

Moreover, the layout design of sensor nodes strongly associates with sensors for transmission and charging. Jiang et al. studied a wireless charger deployment optimization (WCDO) problem [40]. Rao et al. considered how to charge a wireless sensor network with some node deployment restrictions [41]. Feng et al. studied the energy allocation problem of sensor nodes for sensing and transmission [42]. Li et al. studied a dual-functional mobile sink with data gathering and radio frequency (RF) energy harvesting [43]. Lin et al. proposed a power balance aware deployment method to maximize the lifespan of a WRSN [44]. Rout et al. presented a switching algorithm for sensor nodes based on a Markov decision process in a sustainable WRSN [45]. Shu et al. proposed a novel sensor localization design with the time of charge sequences in WRSNs [46]. Najeeb et al. presented a probabilistic algorithm to extend the lifetime of a wireless rechargeable sensor network without exploring the whole network [47]. In fact, the layout design and optimization of wireless sensor nodes is also a very important research subject in a smart factory. If the wireless sensors can be dynamically configured, the factory operators are able to adjust the layout of the sensor nodes according to the machine’s running time, to keep the machine working continuously.

Next, in some special scenarios, researchers have developed some customized network architectures of WRSNs. Erol-Kantarci et al. proposed a sustainable wireless rechargeable sensor network (SuReSense) for long-term monitoring smart grid [21]. Fu et al. studied an optimal movement pattern of the mobile radio frequency identification (RFID) reader to charge an RFID-based wireless rechargeable sensor network [48]. He et al. studied the energy provisioning problem in a wireless rechargeable sensor network, built from the industrial wireless internet service provider (WISP) and commercial RFID readers [14]. Kaswan et al. studied the charging schedule with a single mobile charger in an on-demand WRSN [49]. Wei and Feng et al. proposed an energy-saving traffic scheduling algorithm for hybrid software-defined WRSNs [50,51]. Ding et al. studied the optimal working schemes with plural base stations and wireless energy transfer devices for WRSNs [52]. Lin et al. proposed a temporal-spatial charging scheduling (TSCS) algorithm to minimize the number of dead nodes for on-demand charging framework in a WRSN [53].

Lastly, some joint optimization problems in the complex scenarios have been studied. Han et al. proposed a grid-based joint routing and charging algorithm to balance the energy problem and survival rates of sensor nodes in an industrial WRSN [54]. Jia et al. presented a joint optimization model to maximize both charging efficiency and routing structure in a WRSN [55]. Zhong et al. developed a collaborative wireless energy replenishment and mobile data gathering mechanism [56]. Guo et al. developed an architecture of joint wireless energy provisioning and anchor-point based mobile data collection in WSNs [57]. Zhao et al. developed a multi-functional mobile entity called SenCar, and proposed a collaborative optimization method of mobile energy provisioning and data gathering [58]. Actually, more and more scholars have found only optimizing the charging strategies of wireless sensor nodes is not always effective, and the joint optimizations with consideration of external constraints are more able to satisfy the actual requirements.

In addition, manufacturing scheduling problems are extensively studied in factory operations. In a smart factory, production operations are highly dynamic and flexible, and minimizing the total completion time of all the production tasks is a very key operational objective [18]. In order to shorten the completion time, factory operators should have the capability to reasonably schedule the production tasks and operable machines. It has been proved that manufacturing scheduling problems, essentially belonging to Job shop scheduling problems (JSP), are a class of NP-hard problems [59]. Many models and algorithms have been proposed to obtain a suboptimal solution, such as PICRO (preference-inspired chemical reaction optimization algorithm) [60], HPGA (hybrid PSO-GA algorithm) [61], PRGA-Sched (priority rule-based genetic algorithm scheduling) [62], LCAFS (league championship algorithm with free search) [63], MOGA-TIG (multi-objective genetic algorithm with tabu-enhanced iterated greedy local search strategy) [64], and so on. Though an approximate optimal production plan can be effectively solved by these methods, the availability and reliability of sensor nodes is seldom followed closely. Especially in a smart factory environment, plenty of sensors need to be mounted on the machines, and the availability of sensor nodes is a non-negligible problem. In other words, the suboptimal production plan must be workable in accordance with the availability of sensor nodes, and an unpredictable sensor failure is absolutely not allowed.

In summary, with smart industrial wireless sensor networks being widely applied in smart factories, wireless charging technology provides a good way to replenish energy for SIWSNs, but how to effectively control the charging process closely depends on the actual situation. At present, most studies focus on the application of WRSNs in an open outdoor environment. In these scenarios, wireless rechargeable sensor networks are laid out in a very large space, the distribution of sensor nodes is relatively sparse, the frequency of data acquisition is relatively low, and the energy consumption of sensor nodes is relatively low. Different from the application of WRSNs in a general environment, the complexity and time-effectiveness of an industrial environment cannot be neglected. In fact, industrial wireless sensors are often densely laid out in a workshop with limited space, the frequency of data acquisition may be at the millisecond level on a working machine, and the energy consumption of sensor nodes is relatively high. At the same time, in the case of a given production plan, the associated wireless sensor failures must be prevented during the machine processing some work stage of one workpiece. Charging for the wireless sensors and processing on the associated machines must be separated from Therefore, only maximizing energy provisioning cannot meet the requirements of factory operations, and a framework of multiple constraints joint optimization is worth studying for SIWRSNs in smart factories.

## 3. Preliminaries

In this section, we briefly present an overview of the problem statement and model assumptions.

### 3.1. Problem Statement

In a smart factory [18], the production activities are highly adaptive and flexible. Each machine can process multiple types of workpieces. Material handling between machines mainly relies on a smart logistics system. Production plans can be automatically generated based on customer needs and manufacturing knowledge, and be actively sent to the corresponding fabricating machinery. In the meantime, in order to achieve a series of controllable manufacturing scheduling, manufacturing information should be acquired in real-time by sensor nodes, and the manufacturing process should be rigorously monitored by sensor networks, and the manufacturing status should be synchronously fed back to managers by control centers. In order to more flexibly and extensively collect manufacturing information, SIWRSNs will be applied to the smart factory.

For the purpose of accurately and reliably monitoring manufacturing equipment (e.g., machine tools) in a smart factory, SIWRSNs need to make use of reasonable charging policies to ensure the high-load data acquisition process and prevent sensor nodes from stopping due to a low battery. At the same time, to avoid spatial intersecting and signal interference, charging for sensor nodes can only occur when relevant machines stop working. As a result, we must address two critical problems:

- The first problem is how to reasonably replenish energy for sensor nodes under the condition of insuring continuous machining and avoiding sensors failure, based on actively predicting the available electricity of wireless sensor nodes.
- The second problem is how to effectively assign production tasks to the optional machines under the constraint of operational relationships, and further minimize the processing time of the entire production tasks on the basis of separation between nodes charging and machines processing.

### 3.2. Model Assumptions

Given the complexity of the practical manufacturing processes, we set up some assumptions about this problem and appropriately refine the model. For SIWRSNs, we suppose the communication is very reliable, and the communication errors and retransmissions will not be considered. In addition, because the nodes’ operation is relatively stable in a smart factory environment [18], the variability of nodes’ operations will not be involved. This paper mainly focuses on how to jointly optimize charging strategies and scheduling plans for minimizing the total completion time of all the production tasks in a smart factory as much as possible.

In a smart factory, we suppose the production tasks have some workpieces requiring to be processed on some machines [65]. According to manufacturing requirements, each workpiece must successively go through several workstages, and the sequence of workstages is absolutely unalterable. On each workstage, multiple machines are optional, but only one machine can be selected. In the meantime, some industrial wireless sensors are mounted on these machines, which are used for the timely collection of manufacturing process information. According to the plan of charging scheduling, wireless sensors of low energy can be replenished by portable wireless chargers after the associated machine finishes some workstages. Considering the low power and low cost of portable plug-in wireless charging devices [66], we suppose the wireless charging processes are not restricted by the number and capacity of chargers.

From the above, the detailed model assumptions are as follows:

- Each machine can only process one workstage at most in synchronization.
  Each workstage can only be processed by one machine at most in synchronization.
- The processing sequence of each workpiece must satisfy the requirements of production, and the different workstages of a workpiece cannot coincide.
- Once the workstage starts processing, it cannot be interrupted until processing is completed.
- The workpieces are independent from each other, there is no sequence constraint, and there is no difference between priorities.
- At the beginning, the initial state of all machines is idle.
- At the beginning, all the workpieces arrive at the factory to be processed.
- The transfer time between the different workstages of a workpiece is negligible.
- The working output current of wireless sensors is constant by default.
- The charging input current of wireless sensors is constant by default.
- When the machine is not working, wireless sensors can enter low-power mode, regardless of the energy consumption during this period.
- At the beginning, all the wireless sensors are at full charge.
- Charging for wireless sensors starts with the associated machine finishing one workstage and has stopped working.
- When wireless sensors are charging, the associated machine must shut down.
- Regardless of the capacity loss of the battery, the maximum battery capacity of the wireless sensor is constant.
- The wireless sensor adopts greedy charging such that it charges until it is full.
- The maximum battery capacity of wireless sensors is sufficient to support any workstage.
- The uncertainty of communication and operations on the sensor nodes is negligible.

## 4. Joint Energy Provisioning and Manufacturing Scheduling (J-EPMS)

In this section, we provide the detailed implementations of Joint Energy Provisioning and Manufacturing Scheduling (J-EPMS) in smart industrial wireless rechargeable sensor networks. We first define the symbols and variables required for modeling. Second, we consider how to establish a model of J-EPMS with charging strategies and scheduling plans. Finally, a novel solution algorithm is proposed.

### 4.1. Symbol Definition

Table 1 gives the notations that are defined for building a framework of J-EPMS.

### 4.2. Decision Variables

These decision variables are defined for optimizing the charging strategies and production plans of J-EPMS as given in Table 2.

### 4.3. Model Establishment

With the aim of building a framework of J-EPMS, the battery capacity and charging time of wireless sensors must be taken into consideration in SIWRSNs. Meanwhile, production tasks are restricted by manufacturing techniques and machines’ capabilities in a smart factory [67]. On the basis of previously defined notations, we comprehensively establish an optimization model of J-EPMS. This model mainly includes six types of constraints and an objective function.

First, Sequence Constraints of Workstages are presented as:

(1) $$ST_{ij}\geq FT_{i\left(j-1\right)},\;i=1,\;2,\ldots ,n;j=1,\;2,\ldots ,N_{i},$$

The Sequence Constraints of Workstages highlight that the processing sequence of each workstage cannot be violated. For each workpiece, the latter workstage only begins after the previous one has been completed.

Second, Exclusive Constraints of Workstages are presented as:

(2) $$\overset{m}{\underset{k=1}{\sum }}x_{ijk}=1,\;i=1,\;2,\ldots ,n;j=1,\;2,\ldots ,N_{i},.$$

The Exclusive Constraints of Workstages emphasize that each workstage of the workpieces can only be processed on one machine. In other words, each workstage of all the workpieces must be assigned to one machine.

Third, Processing Time Constraints of Machines are presented as:

(3) $$ST_{ftk}\geq FT_{ijk},\;k=1,\;2,\ldots ,m,$$

where $x_{ijk}=1,\;x_{ftk}=1,\;y_{ftk}=y_{ijk}+1$.

The Processing Time Constraints of Machines highlight that the different workstages assigned to the same machine must be processed by order. The beginning time of the latter workstage must be equal or greater than the finishing time of the previous workstage on the same machine.

Fourth, Deployment Constraints of Wireless Sensors are presented as:

(4) $$\overset{m}{\underset{k=1}{\sum }}d_{rk}=1,r=1,\;2,\ldots ,q,$$

The Deployment Constraints of Wireless Sensors emphasize that each wireless sensor can only be mounted on one machine. However, a machine can hold multiple wireless sensors. In this paper, we suppose the wireless sensors have been mounted on the machines in advance, and the deployment optimization of wireless sensors will not be considered.

Fifth, Battery Capacity Constraints of Wireless Sensors are presented as:

(5) $$\underset{i,j}{\sum }MT_{ijk}\leq \underset{r}{\min}\left(C_{r}/EO_{r}\right),\;k=1,\;2,\ldots ,m,$$

where $P_{ij}\in \left(ps_{k}^{\left(v_{1}\right)},\;ps_{k}^{\left(v_{1}+1\right)},\ldots ,\;ps_{k}^{\left(v_{2}\right)}\right);\;\forall v_{1}\in \left\{v|v=1\;or\;z_{rk}^{\left(v-1\right)}=1\right\};\;z_{rk}^{\left(v_{2}\right)}=1;\;\forall v\in \left(v_{1},v_{2}\right),$ $z_{rk}^{\left(v\right)}=0;d_{rk}=1$.

The Battery Capacity Constraints of Wireless Sensors highlight that the continuous processing time of each machine cannot be greater than the maximum working time of each associated wireless sensor. The continuous processing time of a machine can be calculated by the time interval between charging for the associated wireless sensors. The maximum working time of wireless sensors can be calculated by dividing the battery capacity by the working output current.

Sixth, Charging Time Constraints of Wireless Sensors are presented as:

(6) $$ST_{\lambda \mu }-FT_{\alpha \beta }\geq \underset{r}{\max}\left\{\left(C_{r}-\underset{i,j}{\sum }MT_{ijk}\right)/EI_{r}\right\},\;k=1,\;2,\ldots ,m,$$

where $P_{ij}\in \left(ps_{k}^{\left(v_{1}\right)},\;ps_{k}^{\left(v_{1}+1\right)},\ldots ,\;ps_{k}^{\left(v_{2}\right)}\right);\;\forall v_{1}\in \left\{v|v=1\;or\;z_{rk}^{\left(v-1\right)}=1\right\};\;P_{\alpha \beta }=ps_{k}^{\left(v_{2}\right)},\;z_{rk}^{\left(v_{2}\right)}=1$, $P_{\lambda \mu }=ps_{k}^{\left(v_{2}+1\right)};\;\forall v\in \left(v_{1},v_{2}\right),\;z_{rk}^{\left(v\right)}=0;\;d_{rk}=1$.

The Charging Time Constraints of Wireless Sensors emphasize that the charging time for wireless sensors must be enough to meet the requirement of greedy charging. In other words, when the wireless sensors are arranged to charge at the end of a workstage, the beginning time of the next workstage on the associated machine cannot be earlier than the finishing time of greedy charging. The time for greedy charging for wireless sensors can be calculated by dividing the remaining battery after the continuous processing by the charging input current.

Finally, the objective function was:

(7) $$minT_{max}=min\left(max_{i=1}^{n}WT_{i}\right)$$

On account of the charging time for wireless sensors included in the processing time of the associated workpieces, our optimization objective is simply to minimize the maximum completion time of production tasks. In this model, a continuous timeline is considered, and the maximum completion time of production tasks can be represented by calculating the maximum completion time of all the workpieces.

### 4.4. Solution Algorithm

From the perspective of modeling, this framework of J-EPMS is essentially the joint optimization of flexible manufacturing scheduling and charging schemes for SIWRSNs. As we know, the flexible manufacturing scheduling problem has been proved to be a NP-hard problem [59]. So the theoretical optimal solution of J-EPMS is too hard to be obtained. However, the approximate optimal solutions are also acceptable and meaningful in an industrial context. Due to the advantages of tactical applicability and computational feasibility, evolutionary algorithms are widely studied to search the approximate optimal solutions of these problems in recent years, such as simulated annealing, particle swarm, and genetic algorithms. On account of the validity and reliability of genetic algorithms, genetic algorithms are extensively studied to tackle the flexible manufacturing scheduling problem [68]. However, there exists rapid local convergence and low search efficiency for the classical genetic algorithm. To improve the search efficiency and rapid convergence, quantum genetic algorithm (QGA) is proposed by combining quantum computing with genetic algorithm [69,70]. QGA redefines quantum bits, quantum coding, and quantum superposition to replace classical chromosomal coding with the probability amplitude of quantum bits [71]. Additionally, double chains coding is widely used in solving manufacturing scheduling problems by genetic algorithms. Therefore, for the purpose of effectively obtaining the approximate optimal solutions of J-EPMS, we propose a novel double chains quantum genetic algorithm with Taboo search (DCQGA-TS).

First, in DCQGA-TS, we utilized double chains quantum coding to represent the variant feasible solution of J-EPMS, and the coding scheme is described as:

(8) $$X_{i}=\left(\begin{array}{c} \cos\gamma_{i1}\\ \sin\gamma_{i1} \end{array}\left|\begin{array}{c} \cos\gamma_{i2}\\ \sin\gamma_{i2} \end{array}\right|\begin{array}{c} \cos\gamma_{i3}\\ \sin\gamma_{i3} \end{array}\left|\begin{array}{c} \cdots \\ \cdots \end{array}\right|\begin{array}{c} \cos\gamma_{ij}\\ \sin\gamma_{ij} \end{array}\left|\begin{array}{c} \cdots \\ \cdots \end{array}\right|\begin{array}{c} \cos\gamma_{ih}\\ \sin\gamma_{ih} \end{array}\right),$$

where *i* = 1, 2, 3, …, *g*; *j* = 1, 2, 3, …, *h*; *g* is the population size; *h* is the size of quantum bits and equals the total number of workstages of all workpieces; $X_{i}$ represents the *i*th double-chromosome; $\gamma_{ij}$ represents the *j*th quantum angle of $X_{i}$, $\gamma_{ij}\in \left[0,\;2\pi \right]$. Each double-chromosome $X_{i}$ represents a candidate optimal solution, containing two parallel gene chains, which is described as:

(9) $$X_{i}=\left(\begin{array}{c} X_{i1}\\ X_{i2} \end{array}\right),$$

where $X_{i1}=\left(\cos\gamma_{i1}\left|\cos\gamma_{i2}\right|\cos\gamma_{i3}\left|\cdots \right|\cos\gamma_{ih}\right)$ is defined as the selection chain of all machines and represents the candidate solution of selecting the processing machine for all workpieces; $X_{i2}=\left(\sin\gamma_{i1}\left|\sin\gamma_{i2}\right|\sin\gamma_{i3}\left|\cdots \right|\sin\gamma_{ih}\right)$ is defined as the sequence chain of all workpieces and represents the candidate solution of arranging the processing sequence for all workpieces.

Second, we used a double-chain encoding scheme to map the double-chromosome $X_{i}$ to the solution space. For the gene chain $X_{i1}$, we utilized the linear transformation to map cosine function values into the solution space. The linear transformation formula is described as:

(10) $$X_{i1}^{\left(j\right)}=\lceil X_{i1}^{l}+\frac{\cos\gamma_{ij}+1}{2}\left(X_{i1}^{u}-X_{i1}^{l}\right)\rceil ,$$

where ⌈·⌉ denotes rounding up to an integer; $X_{i1}^{\left(j\right)}$ represents the *j*th value of $X_{i1}$; $X_{i}^{l}=0,\;X_{i}^{u}=m$, where *m* denotes the total number of machines. Therefore, $X_{i1}^{\left(j\right)}$ can take any value between 1 and *m*.

For the gene chain $X_{i2}$, we used a displacement encoding to transform sine function values into the solution space. In the population initialization, we randomly generated a feasible processing sequence of all workpieces for each chain $X_{i2}$ as parent individual *P*. For example, if we have three workpieces and each workpiece has two workstages, {1 2 1 3 2 3} is a feasible processing sequence, where the number denotes the serial number of the workpieces and the frequency of occurrence denotes the workstage of the corresponding workpiece, e.g., the first 1 denotes the first workstage of the first workpiece.

Then we utilized a random number r∈[0, 1] to generate a binary sequence *S* by comparing sine function values with *r*. If ${\left(X_{i2}^{\left(j\right)}\right)}^{2}\geq r$, the *j*th value of *S* is 1; otherwise is 0. On the basis of the parent individual *P* and binary sequence *S*, we could generate a new feasible processing sequence as a child individual *C*. If $S^{\left(j\right)}=1,$ then $C^{\left(j\right)}=P^{\left(j+1\right)}$, where $S^{\left(j\right)}$ denotes the *j*th value of the binary sequence *S*, $C^{\left(j\right)}$ denotes the *j*th value of the child individual *C*, and $P^{\left(j\right)}$ denotes the *j*th value of the parent individual *P*. We got a whole child individual *C* by filling in *C* with the residual values of *P* in turn. In the meantime, the child individual *C* was regarded as the parent individual *P* in the next iteration. For example, if *P* is {1 2 1 3 2 3} and *S* is {1 0 0 1 1 0}, then *C* is {2 1 1 2 3 3}.

Third, considering the fact that the processing time of all the workpieces is definitely much greater than the charging time for SIWRSNs and the charging time of wireless sensor is extremely unstable in a smart factory, Taboo search was applied to search the suitable charging windows for low-energy wireless sensors on the basis of the decoded double-chromosome. The search strategy was to preferentially find a discontinuous time window of the workstage completion to charge the wireless sensors before the wireless sensors’ failure.

Fourth, we used the quantum rotation gate to update the double-chromosome [71], and the rotation gate is described as:

(11) $$U\left(\theta \right)=\left(\begin{array}{cc} \cos\theta & -\sin\theta \\ \sin\theta & \cos\theta \end{array}\right),$$

where U(θ) represents the quantum rotation gate, and *θ* represents the angle of rotation.

The rotation operation is:

(12) $$\left(\begin{array}{cc} \cos\theta & -\sin\theta \\ \sin\theta & \cos\theta \end{array}\right)\times \left(\begin{array}{c} \cos\gamma_{ij}\\ \sin\gamma_{ij} \end{array}\right)=\left(\begin{array}{c} \cos(\gamma_{ij}+\theta )\\ \sin(\gamma_{ij}+\theta ) \end{array}\right),$$

The rotation angle of each quantum bit $\theta_{ij}$was calculated using:

(13) $$\theta_{ij}=\mathrm{sgn}\left(\cos\gamma_{ij}\sin\gamma_{pj}-\cos\gamma_{pj}\sin\gamma_{ij}\right)\times \theta_{0},$$

where $\theta_{0}$ denotes the basic rotation angle; sgn(·) represents the direction of rotation; $\left(\cos\gamma_{pj},\;\sin\gamma_{pj}\right)$ represents the quantum angle of the optimal double-chromosome.

Fifth, to increase the diversity of population, we used a Hadamard gate. The Hadamard gate is described as:

(14) $$\frac{1}{\sqrt{2}}\left(\begin{array}{cc} 1 & 1\\ 1 & -1 \end{array}\right)\times \left(\begin{array}{c} \cos\gamma_{ij}\\ \sin\gamma_{ij} \end{array}\right)=\left(\begin{array}{c} \cos(\frac{\pi }{4}-\gamma_{ij})\\ \sin(\frac{\pi }{4}-\gamma_{ij}) \end{array}\right),$$

Finally, to minimize the maximum completion time of production tasks, we utilized the elite retention strategy to retain the optimal double-chromosome in each iteration. The detailed procedures of DCQGA-TS for J-EPMS are given in Algorithm 1.

**Algorithm 1.** Presents the detailed procedures of DCQGA-TS for J-EPMS.
**Input:** the population size *g*, the maximum number of iterations *MaxIteration*, the basic rotation angle $\theta_{0}$, and the mutation probability $P_{\varepsilon }$.**Step1:** using double chains quantum coding to generate g double-chromosomes, as the initial population;**Step2:** using the double-chain encoding scheme to get the solution space of *g* double-chromosomes;**Step3:** using Taboo search to find the suitable charging windows for low-energy wireless sensors on the basis of the decoded individual, and calculating the maximum completion time $T_{max}$ of each solution with the constraints of battery capacity and charging time;**Step4:** comparing the current optimal solution with the global optimal solution, and retaining the smaller as the global optimal solution;**Step5:** if the number of iterations is equal to *MaxIteration*, then exit and output the global optimal solution; otherwise, go to Step6;**Step6:** calculating the rotation angle *θ*, and using the quantum rotation gate to update the double-chromosome;**Step7:** using the Hadamard gate to make the mutation of double-chromosome on the basis of the mutation probability $P_{\varepsilon }$, and jumping to Step2.


Since the computation complexity of J-EPMS grows exponentially, we proposed the double chains quantum genetic algorithm with Taboo search (DCQGA-TS) to obtain a suboptimal solution. Some studies have proved that genetic algorithms can effectively tackle these kinds of NP-hard problems [60]. Other studies have shown that the quantum genetic algorithm can more effectively and rapidly find a suboptimal solution, compared with the traditional genetic algorithm [72]. By analyzing the detailed procedures of DCQGA-TS, a well-designed double chains quantum coding scheme of the quantum genetic algorithm was proposed for J-EPMS. In order to further enhance algorithm efficiency, we used the Taboo search (TS) strategy to find the suitable charging windows for low-energy wireless sensors based on the decoded double-chromosome. In a word, our proposed algorithm has an advantage over the traditional genetic algorithm for computation efficiency and accuracy.

## 5. Simulations

In this section, we take a production task on multiple machine tools in a smart workshop, for example, to verify the feasibility and efficiency of the proposed DCQGA-TS for J-EPMS. It is important to be noted that these data have been collected and collated from the actual production environment. Since we mainly focus on the validity of the framework and algorithm, how to get these simulated data will not been further described.

For this production task, there were four types of workpieces required to be processed on five machines, and the detailed processing time is given in Table 3. The required quantity of these four types of workpieces was 30, 18, 25, and 20, respectively. There were two wireless sensors mounted on each machine, and the maximum battery capacity of each sensor was 600 mAH. The working output current of each wireless sensor was 15 mA, and the charging input current of each wireless sensor was 300 mA. We suppose that the working current and charging current of each wireless sensor were both constant. Therefore, we can calculate that the maximum working time of each wireless sensor was 40 h, and the maximum charging time of each wireless sensor was 2 h.

**Figure 1:**
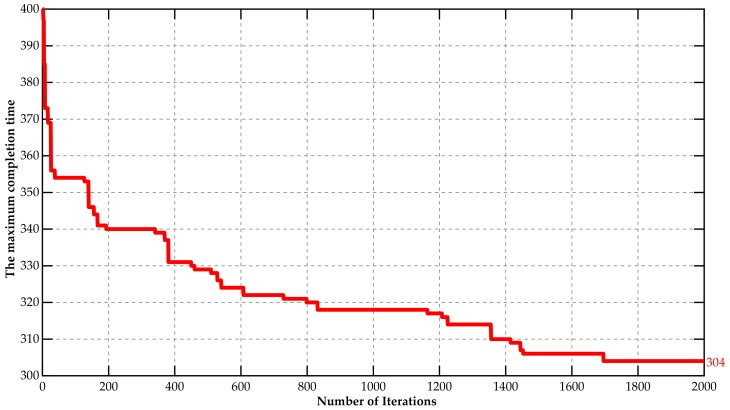
The variation of the maximum completion time optimizing with the constraints of wireless sensors.

**Figure 2:**
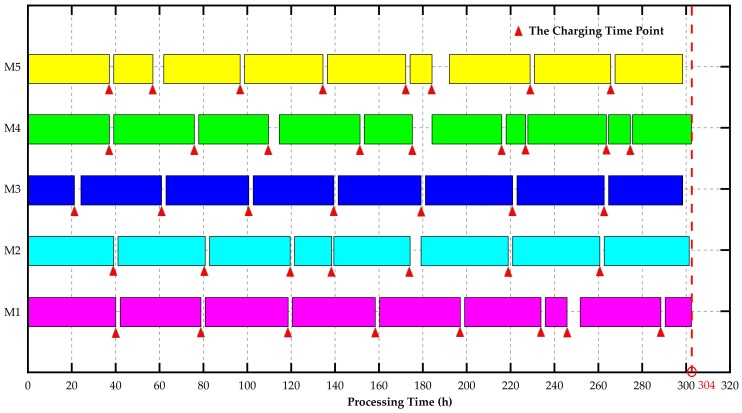
The time distribution of charging for wireless sensors and continuous processing for five machines based on J-EPMS.

On the basis of above data, we implemented a MATLAB program for DCQGA on MATLAB 2017b. The population size *g* was 100; the maximum number of iterations *MaxIteration* was 2000; the basic rotation angle $\theta_{0}$ was 0.01π; the mutation probability $P_{\varepsilon }$ was 0.1. The optimization iteration process is described as in Figure 1. The approximate optimal solution was 304 h after 2000 iterations. Accordingly, the charging strategies and manufacturing scheduling is shown as Figure 2. The simulation results show that the framework of J-EPMS could ensure the separation between nodes charging and machines processing, and the algorithm of DCQGA-TS could optimize the maximum completion time of the entire production task.

**Figure 3:**
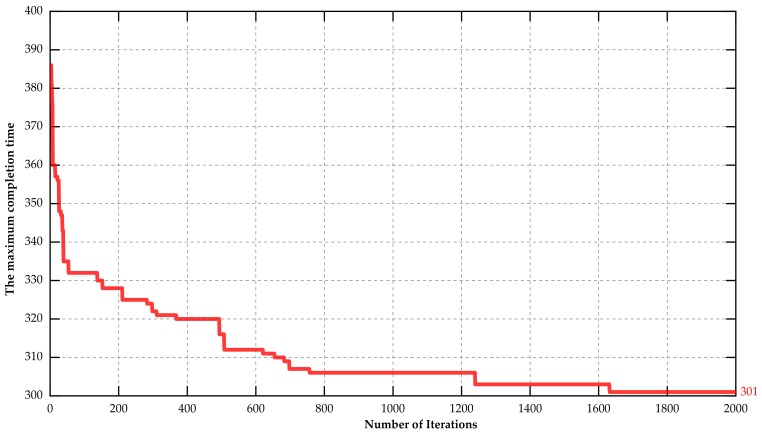
The variation of the maximum completion time optimizing without the constraints of wireless sensors.

**Figure 4:**
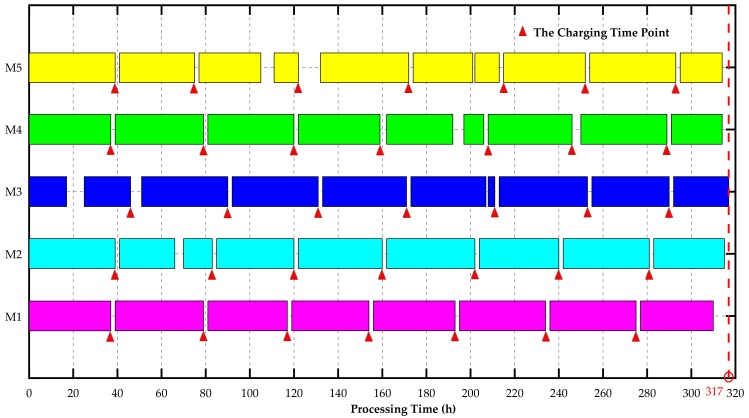
The time distribution of charging for wireless sensors and continuous processing for five machines based on the strategy of passively arranging charging time for wireless sensors with the associated machine having stopped working.

In order to highlight the advantage of the proposed J-EPMS, we also implemented a MATLAB program without the constraints of wireless sensors under the same parameters. The optimization process without sensor constraints is described as Figure 3. Since the charging time of wireless sensors is not considered, the approximate optimal solution of 301 h did not satisfy the practical needs. To avoid the appearance of unexpected processing interruption caused by wireless sensor failures, the wireless sensors should be charged before the batteries run out, with the associated machines stopping working. Therefore, executable scheduling plans involving charging time points and machines processing time is described in Figure 4. The true maximum completion time was 317 h, greater than the approximate optimal solution for J-EPMS.

For the above simulation results, we can come to the following two verifications. First, our developed framework of J-EPMS could ensure machines are not unexpectedly interrupted by wireless sensor failures. Second, our proposed algorithm of DCQGA-TS for J-EPMS could effectively shorten the maximum completion time of the entire production task. Though the two simulated scenarios are not very complex, we could verify that the joint optimization framework of J-EPMS was capable of describing the complex relationship between the charging strategies and manufacturing scheduling. At the same time, the comparison between the solved scheduling plans also confirmed that the proposed algorithm was able to obtain a suboptimal solution based on the calculation results of DCQGA-TS for J-EPMS. In order to more effectively deal with different requirements of factory operations, some complex simulations are intensively focused in our future works.

## 6. Discussion and Conclusions

In this paper, we were concerned about whether factory operators can keep machines with some wireless sensors undergoing high-intensity work for long periods of time by effectively arranging the processing and replenishing energy sequence for SIWRSNs in a smart factory environment. We developed a joint framework of J-EPMS to jointly optimize charging strategies and manufacturing scheduling for minimizing the total completion time of all the production tasks as much as possible. To verify the feasibility of providing continuous factory operations, we set up some assumptions about this problem and appropriately refined the framework. The simulated results of our proposed algorithm of DCQGA-TS for J-EPMS have demonstrated the correctness and practicability of our idea. However, some important factors are not further discussed in the framework, such as communication errors, uncertain nodes’ operations, and the capacity loss of the battery. In some smart factory contexts, the frequent communication errors and retransmissions lead to rapid power consumption, and the uncertain nodes’ operations cause the unavailability of the initial production plan. A dynamic joint optimization framework will be intensively studied in future works.

In summary, we present that it is necessary to apply SIWRSNs in a smart factory environment. In order to effectively replenish energy for SIWRSNs, we have studied how to jointly optimize the charging strategies of wireless sensors and scheduling plans of machines running for minimizing the total completion time of the entire production tasks in a smart factory. We established a collaborative optimization framework of Joint Energy Provisioning and Manufacturing Scheduling (J-EPMS). In this framework, we concluded six types of constraints and an objective function. Since the theoretical optimal solution of J-EPMS is difficult to obtain, we proposed a novel double chains quantum genetic algorithm with Taboo search (DCQGA-TS). The simulation results with DCQGA-TS showed that the framework of J-EPMS can contribute to the reduction of the unpredictable interruptions by wireless sensor failures in production processes. At the same time, DCQGA-TS can decrease the maximum completion time of all the production tasks. In a word, our proposed J-EPMS and DCQGA-TS can contribute to realizing the large-scale applications of smart industrial wireless sensor networks in smart factories.

## Figures and Tables

**Table 1:** List of Notations for J-EPMS.

Notation	Definition
*n*	The total number of workpieces
$W_{i}$	The *i*th workpiece, *i* = 1, 2, …, *n*
$N_{i}$	The number of workstages for the workpiece $W_{i}$
*m*	The total number of machines
$M_{k}$	The *k*th machine, *k* = 1, 2, …, *m*
*q*	The total number of wireless sensors
$S_{r}$	The *r*th wireless sensor, *r* = 1, 2, …, *q*
$P_{ij}$	The *j*th workstage of the workpiece $W_{i}$, *j* = 1, 2, …, $N_{i}$
$MT_{ijk}$	The processing time of the workstage $P_{ij}$ on the machine $M_{k}$
$ST_{ij}$	The beginning time of the workstage $P_{ij}$
$FT_{ij}$	The finishing time of the workstage $P_{ij}$
$WT_{i}$	The completion time of the workpieces $W_{i}$, *i* = 1, 2, …, *n*
$C_{r}$	The battery capacity of the wireless sensor$S_{r}$, *r* = 1, 2, …, *q*
$EO_{r}$	The working output current of the wireless sensor $S_{r}$, *r* = 1, 2, …, *q*
$EI_{r}$	The charging input current of the wireless sensor $S_{r}$, *r* = 1, 2, …, *q*
$T_{max}$	The maximum completion time of the entire production tasks

**Table 2:** List of Decision Variables for J-EPMS.

Variables	Definitions
$x_{ijk}$	If the workstage $P_{ij}$ is processed on the machine $M_{k}$, then $x_{ijk}=1$; otherwise $x_{ijk}=0$
$y_{ijk}$	The sequence number of the workstage $P_{ij}$ processed on the machine $M_{k}$
$ps_{k}$	The processing sequence of the workstage $P_{ij}$ processed on the machine $M_{k}$
$ps_{k}^{\left(v\right)}$	The *v*th value of the processing sequence $ps_{k}$ indicates the workstage $P_{ij}$ is processed on machine $M_{k}$, where $y_{ijk}=v$
$d_{rk}$	If the wireless sensor $S_{r}$ is mounted on the machine $M_{k}$, then $d_{rk}=1$; otherwise $d_{rk}=0$
$z_{rk}^{\left(v\right)}$	If the *v*th workstage $P_{ij}$ of machine $M_{k}$ finishes and the associated wireless sensor $S_{r}$ starts charging at once, then $z_{rk}^{\left(v\right)}=1$; otherwise $z_{rk}^{\left(v\right)}=0$

**Table 3:** List of the Processing Time of Four Workpieces on Five Machines.

Workpieces	Workstages	M1	M2	M3	M4	M5
W1	O11	1	3	4	1	3
	O12	3	8	2	1	10
	O13	3	5	4	7	4
	O14	4	1	1	3	1
W2	O21	2	3	9	3	6
	O12	9	1	2	5	7
	O13	8	6	3	5	6
W3	O31	4	5	8	1	2
	O32	10	7	3	5	9
	O33	6	5	6	10	7
	O34	2	2	5	8	4
	O35	8	3	7	4	5
W4	O41	7	6	4	7	3
	O42	5	3	7	9	2
	O43	4	5	9	3	6
